# Comparison of radiological and clinical outcomes of 3D-printed artificial vertebral body with Titanium mesh cage in single-level anterior cervical corpectomy and fusion: A meta-analysis

**DOI:** 10.3389/fsurg.2022.1077551

**Published:** 2023-01-11

**Authors:** Haiyang Cheng, Gan Luo, Dan Xu, Yuqiao Li, Ziqi Wang, Houzhi Yang, Yang Liu, Yutao Jia, Tianwei Sun

**Affiliations:** ^1^ Graduate School of Tianjin Medical University, Tianjin, China; ^2^ Department of Spinal Surgery, Tianjin Union Medical Center, Tianjin, China; ^3^ School of Medicine, Nankai University, Tianjin, China

**Keywords:** 3D-printed artificial vertebral body, anterior cervical corpectomy and fusion, titanium mesh cage, C2–C7 cobb angle, visual analog scale (VAS) scores, Japanese orthopedic association (JOA) scores, meta-analysis

## Abstract

**Propose:**

This meta-analysis aimed to determine whether 3D-printed artificial vertebral body have superior clinical and radiographic outcome than Titanium Mesh Cage(TMC) in single-level anterior cervical corpectomy and fusion.

**Methods:**

A comprehensive search of the PubMed, Embase, Cochrane Library, Web of Science, and CNKI (China National Knowledge Infrastructure) databases was conducted to find randomized control trials (RCTs) or cohort studies that compared 3D-printed artificial vertebral body with conventional Titanium Mesh Cage (TMC) in single-level anterior cervical corpectomy and fusion (SL-ACCF). Operation time; intraoperative blood loss; subsidence of vertebral body; preoperative, and final follow-up C2–C7 Cobb angle, Japanese Orthopedic Association (JOA) scores, and Visual Analog Scale(VAS) scores were collected from eligible studies for meta-analysis.

**Results:**

We included 6 cohort studies with 341 patients. The results of the meta-analysis showed that the 3D group has a shorter operation time than the traditional TMC group(*p* = 0.04) and the TMC group had more severe subsidence(≥3 mm) of vertebral body than the 3D group(*p* < 0.0001). And the cervical C2–C7 Cobb angle in the 3D group was larger than that in the TMC group at the final follow-up.

**Conclusion:**

This meta-analysis demonstrates that 3D-printed artificial vertebral body is superior to traditional TMC in reducing the operation time and maintaining the postoperative vertebral height and restoring sagittal balance to the cervical spine in single-level anterior cervical corpectomy and fusion.

## Introduction

Recently, the incidence of cervical spondylotic myelopathy (CSM) is increasing (1, 2). Anterior Cervical Corpectomy and Fusion (ACCF) possesses the advantages of complete exposure and sufficient decompression, so it is extensively used in the treatment of a variety of cervical-related diseases (3–7). Autogenous iliac crest, fibula, allogeneic bone, and interbody fusion cages are often implanted in the cervical vertebral body to restore the stability of the anterior column. Autologous bone transplantation will not only extend the operation time, but also cause certain injury and complications (8–11). Titanium Mesh Cage(TMC) with autologous bone or hydroxyapatite artificial bone block has gradually become the most commonly used in ACCF implant materials (12), but TMC, while avoids donor site injury and complication, however, has some disadvantages, such as no close contact between bone and material interface and displacement and subsidence of the prosthesis (9, 13–15). Lu (16) et al. reported 3D-printed adaptive titanium mesh cage for the treatment of CSM and Ossification of the Posterior Longitudinal Ligament (OPLL), showing that 3D-printed artificial vertebral body has good clinical and imaging results in single-level anterior cervical corpectomy and fusion (SL-ACCF). However, due to the short clinical time of D-printed artificial vertebral body, there is a lack of high evidence-based medical evidence on efficacy and safety. This meta-analysis was designed to assess the clinical and radiographic outcomes of 3D-printed artificial vertebral body compared to Titanium Mesh Cage in single-level anterior cervical corpectomy and fusion.

## Methods

The Preferred Reporting Items for Systematic Reviews and Meta-Analyses (PRISMA) statement (17) was used as guidance for our systematic review and meta-analysis.

### Search strategy and study selection

Two investigators independently searched the following databases (inception to Oct 2022): PubMed, Embase, Cochrane Library, Web of Science, and CNKI (China National Knowledge Infrastructure) databases. The electronic search strategy used the following keywords: “Printing, Three-Dimensional”, “Titanium Mesh Cage”, and “anterior cervical corpectomy and fusion”. The search terms were adjusted according to the characteristics of each database, and we also examined the reference lists of the screened full-text studies to identify additional trials that might be eligible. And a third reviewer was consulted when the two reviewers could not reach a consensus.

### Selection strategy

The inclusion and exclusion criteria for this study followed the PICOS principle. (1) Participants: Patients with Cervical Spondylotic Myelopathy (CSM) or Ossification of the Posterior Longitudinal Ligament (OPLL) or other cervical spondylosis, who need to be treated with single-level anterior cervical corpectomy and fusion (SL-ACCF). (2) Intervention and Comparison: In the operation procedure, the implant is the Titanium Mesh Cage (TMC) or the 3D-printed artificial vertebral body, and no implants other than Titanium Mesh Cage (TMC) or 3D-printed artificial vertebral body and titanium plates were used during the procedure. (3) Outcomes: The study should include at least one of the following data: Operation time, intraoperative blood loss, subsidence of vertebral body; preoperative, and final follow-up C2–C7 Cobb angle, Japanese Orthopedic Association (JOA) scores, or Visual Analog Scale (VAS) scores. (4) Study design: Observational studies and randomized control trials were eligible. Surgery with more than one level, case reports, case series, commentaries, practice guidelines, systematic reviews and meta-analysis were excluded. In addition, duplicate studies with the same cohort or studies considered by consensus to be of low quality were excluded.

### Data extraction

Data were extracted from the included studies as follows: (1) study design: ﬁrst author, publication region, publication time, and study type; (2) sample demographics: number of patients, age, sex, and disease diagnosis; (3) surgery details: Type of implants and their details, operation time, intraoperative blood loss; (4) analysis variables: Severe subsidence of vertebral body; operation time, intraoperative blood loss, preoperative, and final follow-up C2–C7 Cobb Angle, Japanese Orthopedic Association (JOA) scores, and Visual Analog Scale(VAS) scores.

### Assessment of risk of bias

Two reviewers evaluated bias risk in the cohort studies using the Newcastle-Ottawa scale (18). Sensitivity analysis was performed by excluding a single study of each study in turn and reanalyzing the data. Publication bias was analyzed qualitatively by funnel plot.

### Statistical analysis

The continuous variables were estimated by weighted mean difference (WMD), and dichotomous variables were estimated by using odds ratios (ORs) with 95% confidence intervals (CIs). The statistical heterogeneity of the pooled results was determined using the *I*² statistic. For this meta-analysis, we used the fixed-effect model when *I*² was greater than 50%, and if *I*²was less than 50%, a random-effect model was applied. The meta-analysis results were considered statistically significant when the *p* value < 0.05. The meta-analysis was performed using Review Manager 5.4 (Revman, The Cochrane Collaboration, Oxford, The UK).

## Results

### Search results

**Figure 1:**
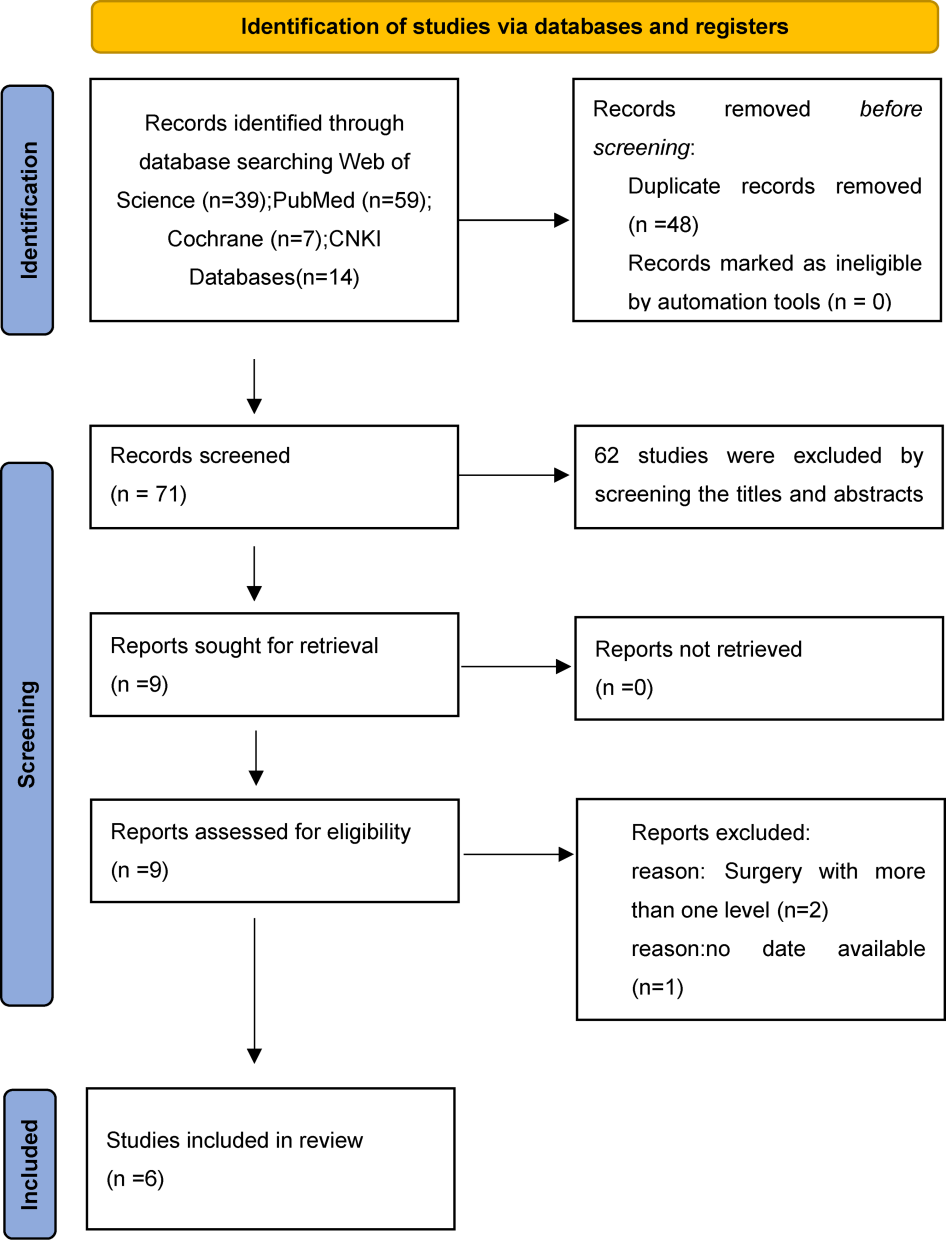
PRISMA flowchart.

A total of 129 articles from PubMed, Web of science, the Cochrane Library, and CNKI were initially identiﬁed. The exact number of articles identiﬁed in each database is as follows: PubMed (*n* = 59), Web of Science (*n* = 39), the Cochrane library (*n* = 7), CNKI (*n* = 14). 48 articles were excluded because of duplication, and 73 studies were excluded by screening the titles and abstracts for: irrelevant studies, case reports, non-comparative studies and review articles. Leaving 7 articles that underwent a comprehensive full-text analysis. Finally, 6 studies (19–24) were included in the ﬁnal meta-analysis. The ﬂow chart used for the new systematic review according to PRISMA 2020 is shown in Figure 1.

### Study characteristics and risk of bias

A total of 341 patients were enrolled in the 6 studies. The 3D-printed artificial vertebral body included 150 patients, and the TMC group included 164 patients. The characteristics of the included studies are presented in Table 1. The quality of included studies was evaluated according to the Newcastle-Ottawa scale, with scores above 7 (including 7) of high quality, and the evaluation results are shown in Table 2.

**Table 1:** Demographics and characteristics of included studies.

Author	Year	Country	Study Design	Operation method	Diagnosis	3D-printed artificial vertebral body		3D group /TMC group	
						Material	Porosity	Sample size (*n*)	Age (mean, year)
Li	2022	China	Cohort studies	Single-level ACCF	OPLL	/	68 ± 5.3%	28/29	52.67/51.51
Han	2022	China	Cohort studies	Single-level ACCF	CSM	/	68 ± 5.3%	25/25	49.10/48.80
Wang	2021	China	Cohort studies	Single-level ACCF	CSM	TA3	NA	30/30	64.03/64.92
Tao	2020	China	Cohort studies	Single-level ACCF	/	Ti6AI4V	80%	20/31	58.83/59.17
Feng	2020	China	Cohort studies	Single-level ACCF	CSM	Ti6AI4V	71%	20/20	55.20/53.80
Zang	2017	China	Cohort studies	Single-level ACCF	CSM/OPLL	Ti6AI4V	NA	27/29	66.25/64.79

CSM, Cervical spondylotic myelopathy; OPLL, Ossification of the posterior longitudinal ligament; ACCF, Anterior cervical corpectomy and fusion.

**Table 2:** Newcastle-Ottawa scale for observational studies.

Author	Year	Selection	Comparability	Outcomes	Quality judgment
Li	2022	4	2	3	9
Han	2022	4	2	2	8
Tao	2020	4	2	3	9
Zang	2017	4	2	3	9
Feng	2020	4	2	2	8
Wang	2021	4	2	3	9

Selection: (1) representativeness of the exposed cohort, (2) selection of the nonexposed cohort, (3) ascer tainment of exposure and (4) demonstration that outcome of interest was not present at the start of stud.

Comparability: comparability of cohorts on the basis of the design or analysis.

Outcomes: (1) assessment outcome, (2) was follow-up long enough for outcomes to occur, (3) adequacy of follow-up of cohorts (≥1 years).

NOS scores ≥ 7 indicate a high-quality study.

## Meta-Analysis results

### Preoperative cervical C2–C7 Cobb angle, VAS scores and JOA scores

**Figure 2:**
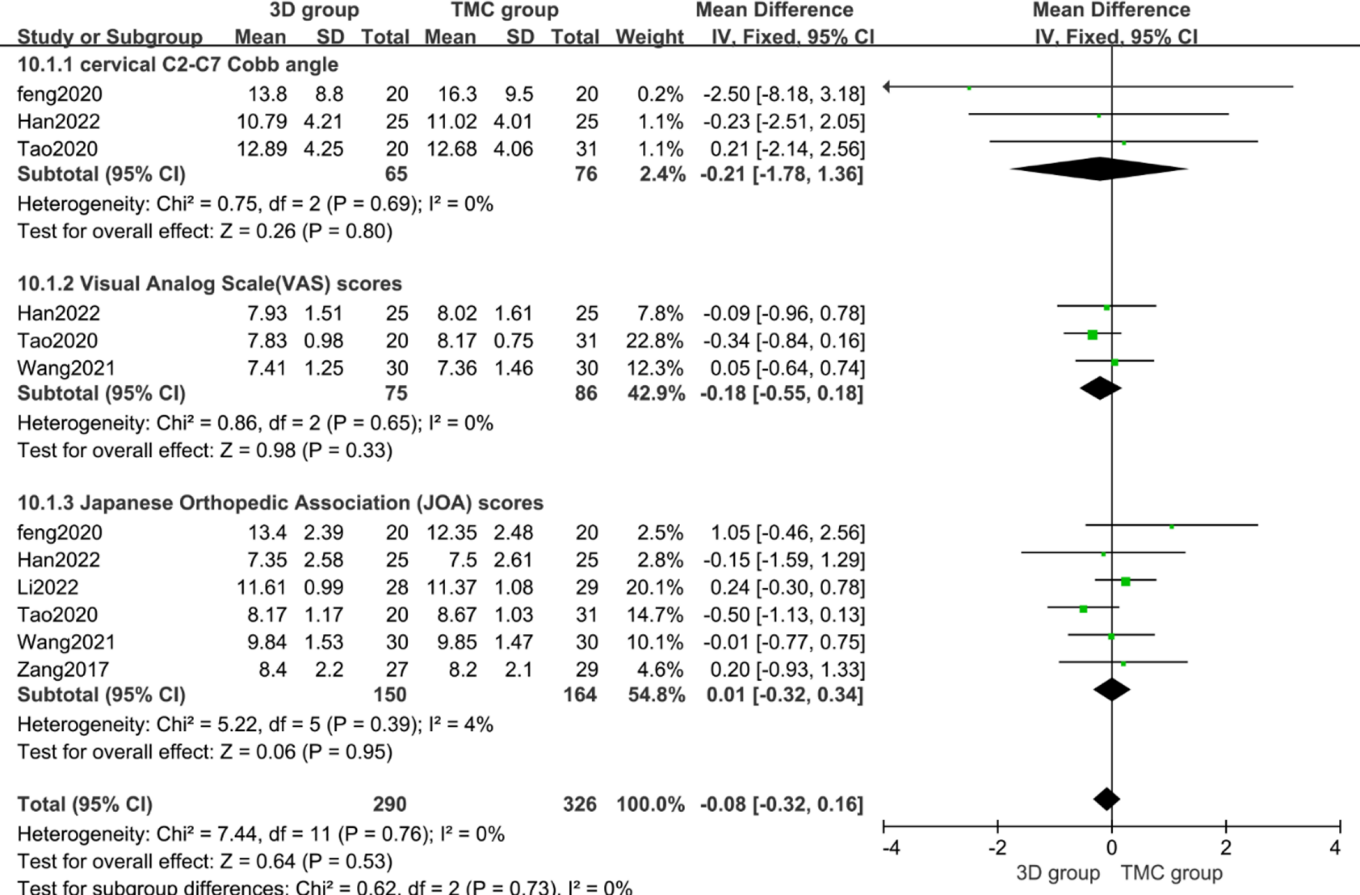
Weighted mean difference of preoperative cervical C2–C7 Cobb angle, Visual Analog Scale (VAS) scores, Japanese Orthopedic Association (JOA) scores between the 3D group and the TMC group. ***SD*** standard deviation, ***CI*** confidence interval, ***IV*** inverse variance.

Preoperative evaluation indicators indicate the baseline level of patients before surgery, three studies (*n* = 141 patients; 65 in the 3D group and 76 in the TMC group) provided the preoperative cervical C2–C7 Cobb angle, three studies (*n* = 161 patients; 75 in the 3D group and 86 in the TMC group) offered Visual Analog Scale(VAS) scores and six studies(*n* = 314 patients; 150 in the 3D group and 164 in the TMC group) reported the preoperative Japanese Orthopedic Association (JOA) scores. No statistically significant difference was observed in the above indicators between the 3D group and TMC group, Combined effect value and heterogeneity test results: Preoperative cervical C2–C7 Cobb angle: *P* = 0.80, WMD −0.21 [−1.78, 1.36], Heterogeneity: Chi² = 0.75, df = 2 (*P* = 0.69); *I*² = 0%; Preoperative Visual Analog Scale(VAS) scores: *P* = 0.33, WMD −0.18 [−0.55, 0.18], Heterogeneity: Chi² = 0.86, df = 2 (*P* = 0.62); *I*² = 0%; and preoperative Japanese Orthopedic Association (JOA) scores: *P* = 0.95, WMD 0.01 [−0.32, 0.34], Heterogeneity: Chi² = 5.22, df = 5 (*P* = 0.39); *I*² = 4% [Figure 2]. which indicated that the included studies did not differ significantly between the 3D group and TMC group at baseline and that the observations were comparable.

### Operation time and intraoperative blood loss

**Figure 3:**
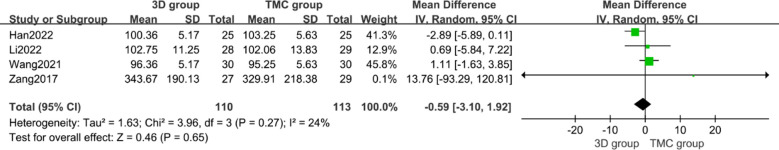
Weighted mean difference of intraoperative blood loss between the 3D group and the TMC group. ***SD*** standard deviation, ***CI*** confidence interval, ***IV*** inverse variance.

**Figure 4:**
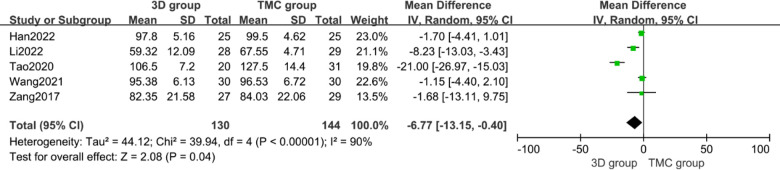
Weighted mean difference of operation time between the 3D group and the TMC group. ***SD*** standard deviation, ***CI*** confidence interval, ***IV*** inverse variance.

Operation time and intraoperative blood loss were used for evaluating the surgical trauma. Five studies (*n* = 274 patients; 130 in the 3D group and 144 in the TMC group) provided operation time and four studies (*n* = 223 patients; 110 in the 3D group and 113 in the TMC group) provided intraoperative blood loss. No statistically significant difference exists in intraoperative blood loss between the two groups (*P* = 0.65, WMD −0.59 [−3.10, 1.92], Heterogeneity: Tau² = 1.63; Chi² = 3.96, df = 3 (*P* = 0.27); *I*² = 24%) [Figure 3]. In contrary, the 3D group showed significantly shorter operation time compared with TMC group (*p* = 0.04, WMD −6.77 [−13.15, −0.40], Heterogeneity: Tau² = 44.12; Chi² = 39.94, df = 4 (*P* < 0.00001); *I*² = 90%) [Figure 4], It may indicate that 3D-printed artificial vertebral body can shorten the operation time compared with conventional Titanium Mesh Cage(TMC), but there is no significant difference in the amount of blood loss during surgery.

### Severe subsidence of vertebral body

**Figure 5:**
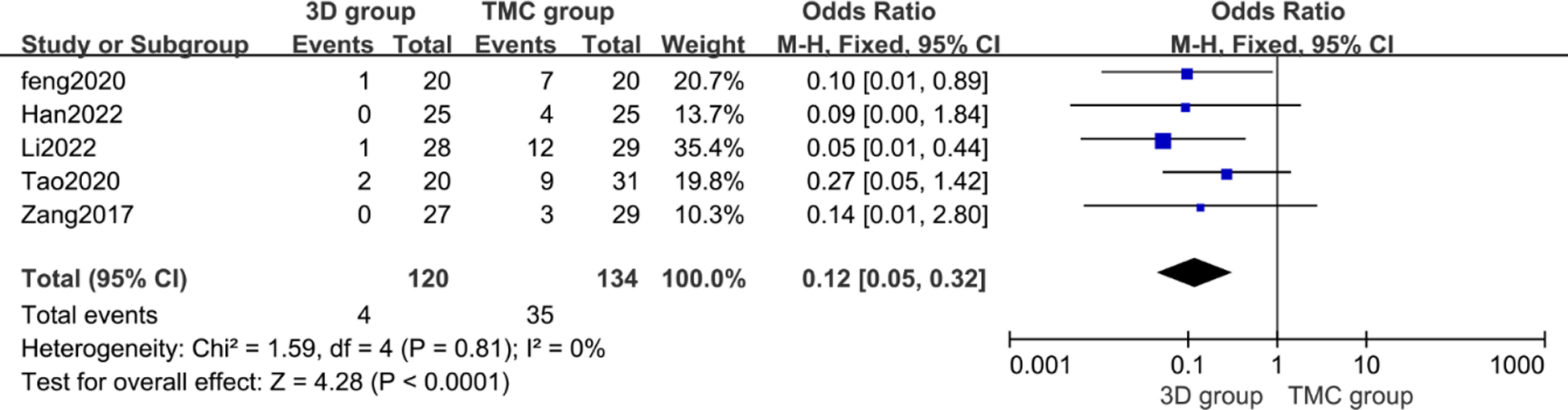
Odds ratio of severe subsidence of vertebral body between the 3D group and the TMC group. ***CI*** confidence interval, ***M-H*** Mantel-Haenszel.

Vertebral subsidence was used to assess the ability of the implant to maintain vertebral height. All included articles defined severe subsidence as a decrease of more than 3 mm in the height of the fused segment during the follow-up period after surgery compared with that immediately after surgery (25, 26). A total of 5 studies reported the severe subsidence of vertebral body (*n* = 254 patients; 120 in the 3D group and 134 in the TMC group). Statistically significant difference was observed in 3D group and TMC group (*p* < 0.0001, OR = 0.12 [0.05, 0.32], Heterogeneity: Chi² = 1.59, df = 4 (*P* = 0.81); *I*² = 0%) [Figure 5]. The incidence of severe subsidence of vertebral body in the 3D group was significantly lower than that in the TMC group, indicating that 3D-printed artificial vertebral body performed significantly better than traditional TMC in maintaining vertebral body height after operation.

### Final follow-up VAS scores and JOA scores

**Figure 6:**
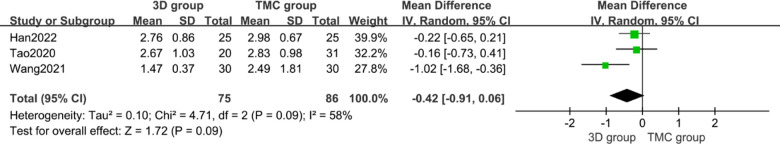
Weighted mean difference of final follow-up Visual Analog Scale (VAS) scores between the 3D group and the TMC group. ***SD*** standard deviation, ***CI*** confidence interval, ***IV*** inverse variance.

**Figure 7:**
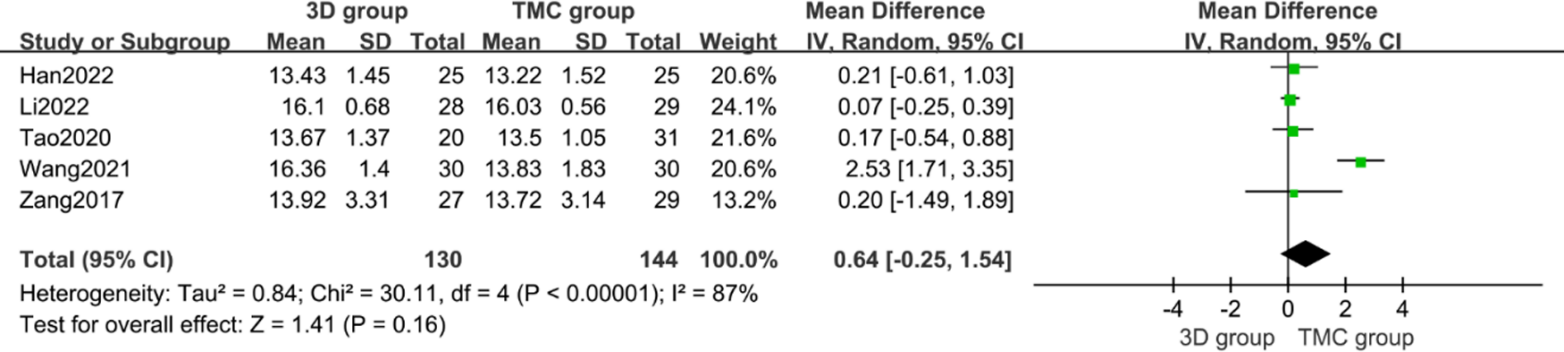
Weighted mean difference of final follow-up Japanese Orthopedic Association (JOA) scores between the 3D group and the TMC group. ***SD*** standard deviation, ***CI*** confidence interval, ***IV*** inverse variance.

The last follow-up time was defined as 1 year after surgery for all included studies. There studies (*n* = 161 patients; 75 in the 3D group and 86 in the TMC group) provided final follow-up VAS scores and four studies (*n* = 274 patients; 130 in the 3D group and 144 in the TMC group) reported the final follow-up JOA scores. There was no statistically significant difference between the two groups regarding final follow-up VAS scores (*P* = 0.98, WMD = −0.42 [−0.91, 0.06], Heterogeneity: Tau² = 0.10; Chi² = 4.71, df = 2 (*P* = 0.09); *I*² = 58%) [Figure 6] and final follow-up JOA scores (*P* = 0.16, WMD = 0.64 [−0.25, 1.54], Heterogeneity: Tau² = 0.84; Chi² = 30.11, df = 4 (*P* < 0.00001); *I*² = 87%) [Figure 7]. Although Japanese Orthopedic Association (JOA) scores and Visual Analog Scale (VAS) scores improved in the last follow-up in each study compared with preoperative assessment, there was no significant difference in the improvement between the two groups.

### Final follow-up cervical C2–C7 Cobb angle

**Figure 8:**
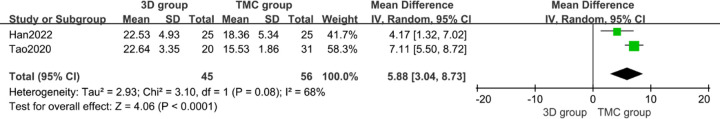
Weighted mean difference of final follow-up cervical C2–C7 Cobb angle between the 3D group and the TMC group. ***SD*** standard deviation, ***CI*** confidence interval, ***IV*** inverse variance.

Similarly, the last follow-up time in the included studies was 1 year after surgery. Two studies (*n* = 101 patients; 45 in the 3D group and 56 in the TMC group) provided the final follow-up cervical C2–C7 Cobb angle. There was statistically significant difference in cervical C2–C7 Cobb angle between the 3D group and TMC group at final follow-up cervical C2–C7 Cobb angle (*P* < 0.0001,WMD 5.88 [3.04, 8.73], Heterogeneity: Tau² = 2.93; Chi² = 3.10, df = 1 (*P* = 0.08); *I*² = 68%) [Figure 8], The results showed that the cervical C2–C7 Cobb angle in the 3D group was larger than that in the TMC group at the final follow-up.

### Heterogeneity and sensitivity analysis

The results of this analysis showed that the heterogeneity of the operation time, final follow-up VAS scores, JOA scores and cervical C2–C7 Cobb angle was high. A random-effect model was used to partially eliminate the effect of heterogeneity, but the results showed that the heterogeneity was still high. After removing one of the included studies for each index (final follow-up VAS scores, JOA scores and cervical C2–C7 Cobb angle), we found that heterogeneity did not decrease significantly, but it did not affect the results, this shows that the results of this meta-analysis are relatively reliable. When it comes to operation time, when we removed each included article separately, the combined effect value shows a significant change or even no significance. Therefore, the conclusion that the 3D group had a shorter operation time than the TMC group is not reliable, and the large heterogeneity may come from the technical level of the surgeons.

### Bias analysis

**Figure 9:**
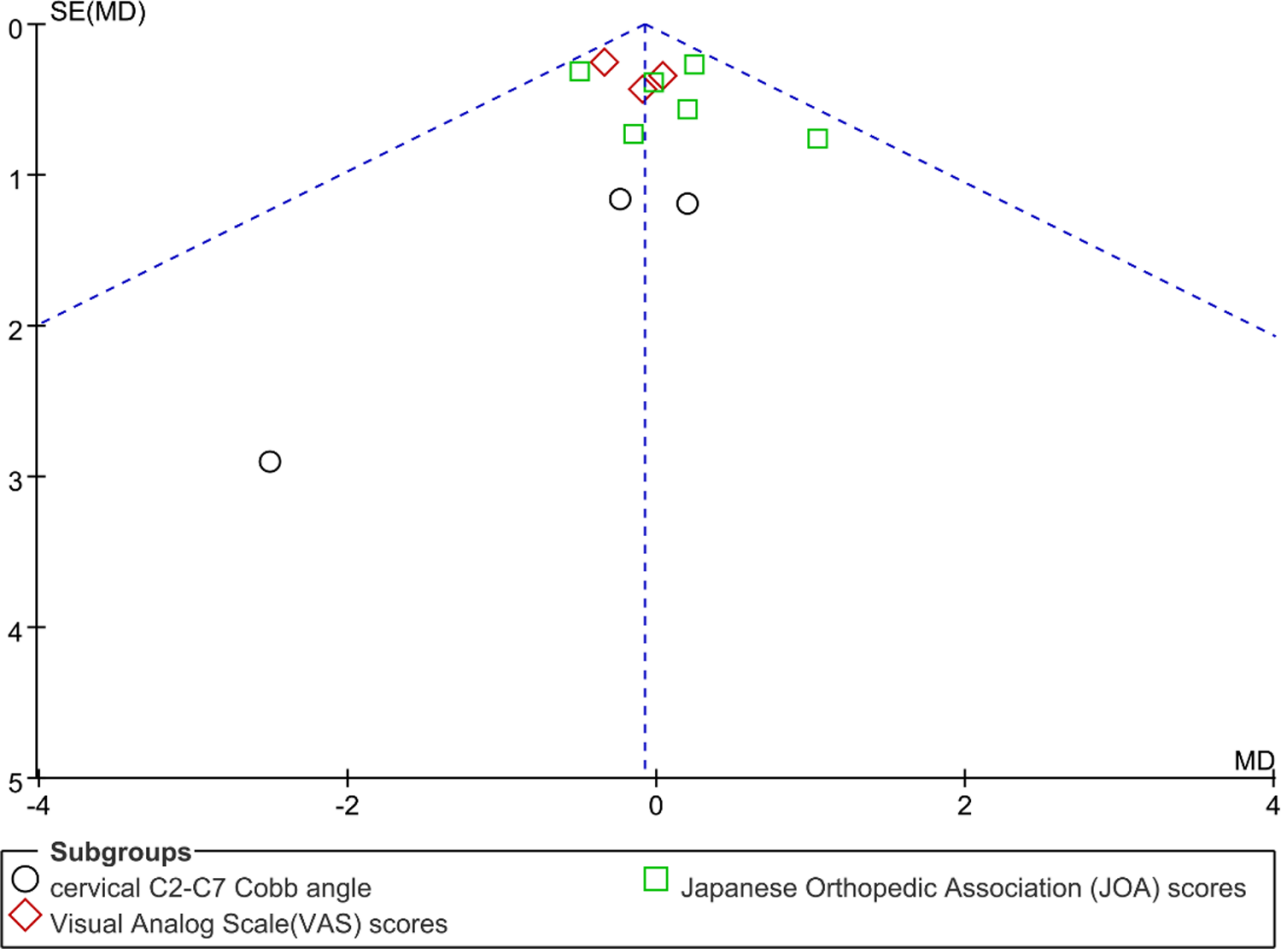
Funnel plot of preoperative cervical C2–C7 Cobb angle, Visual Analog Scale (VAS) scores, Japanese Orthopedic Association (JOA) scores.

**Figure 10:**
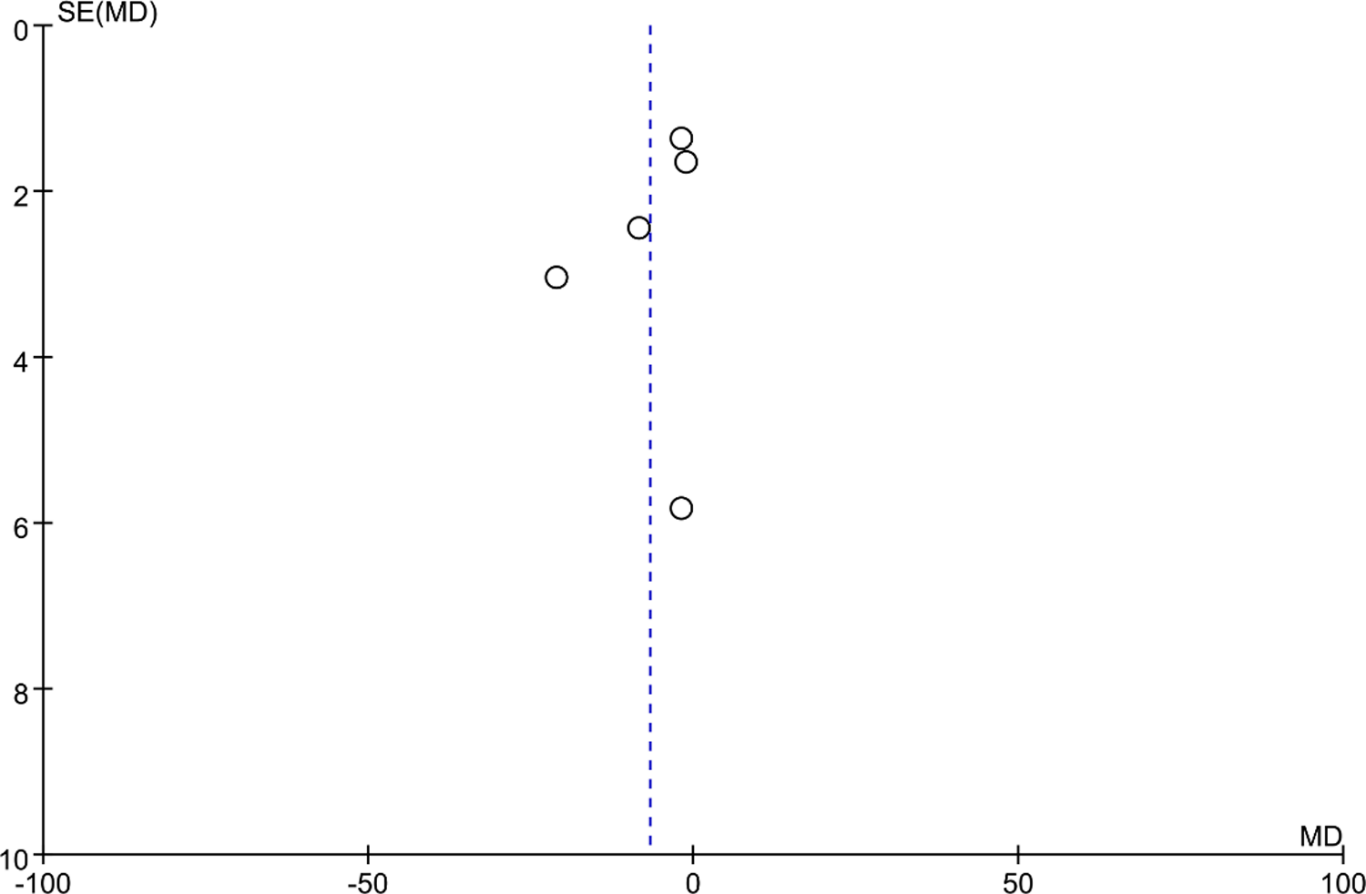
Funnel plot of operation time.

**Figure 11:**
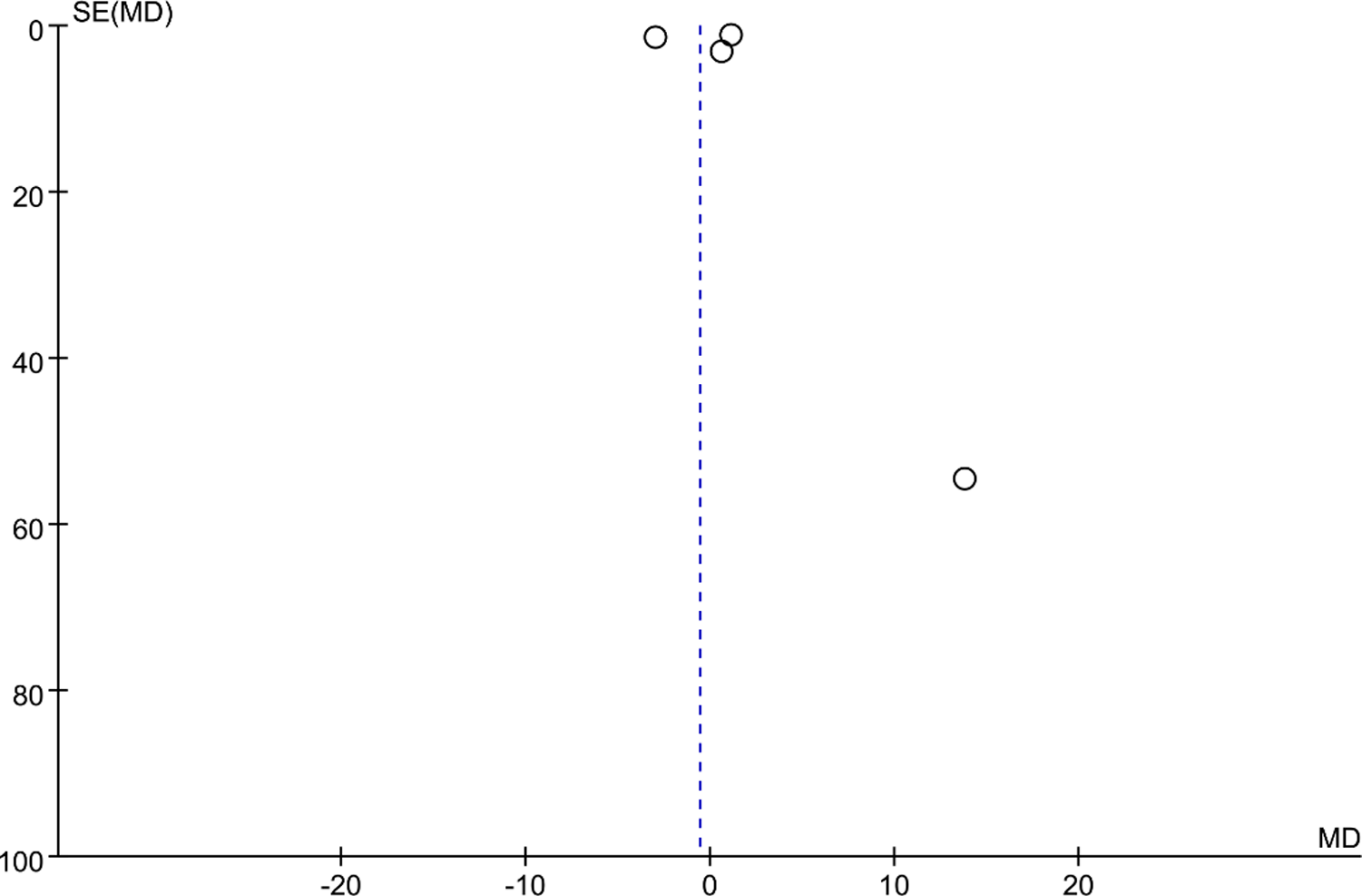
Funnel plot of intraoperative blood loss.

**Figure 12:**
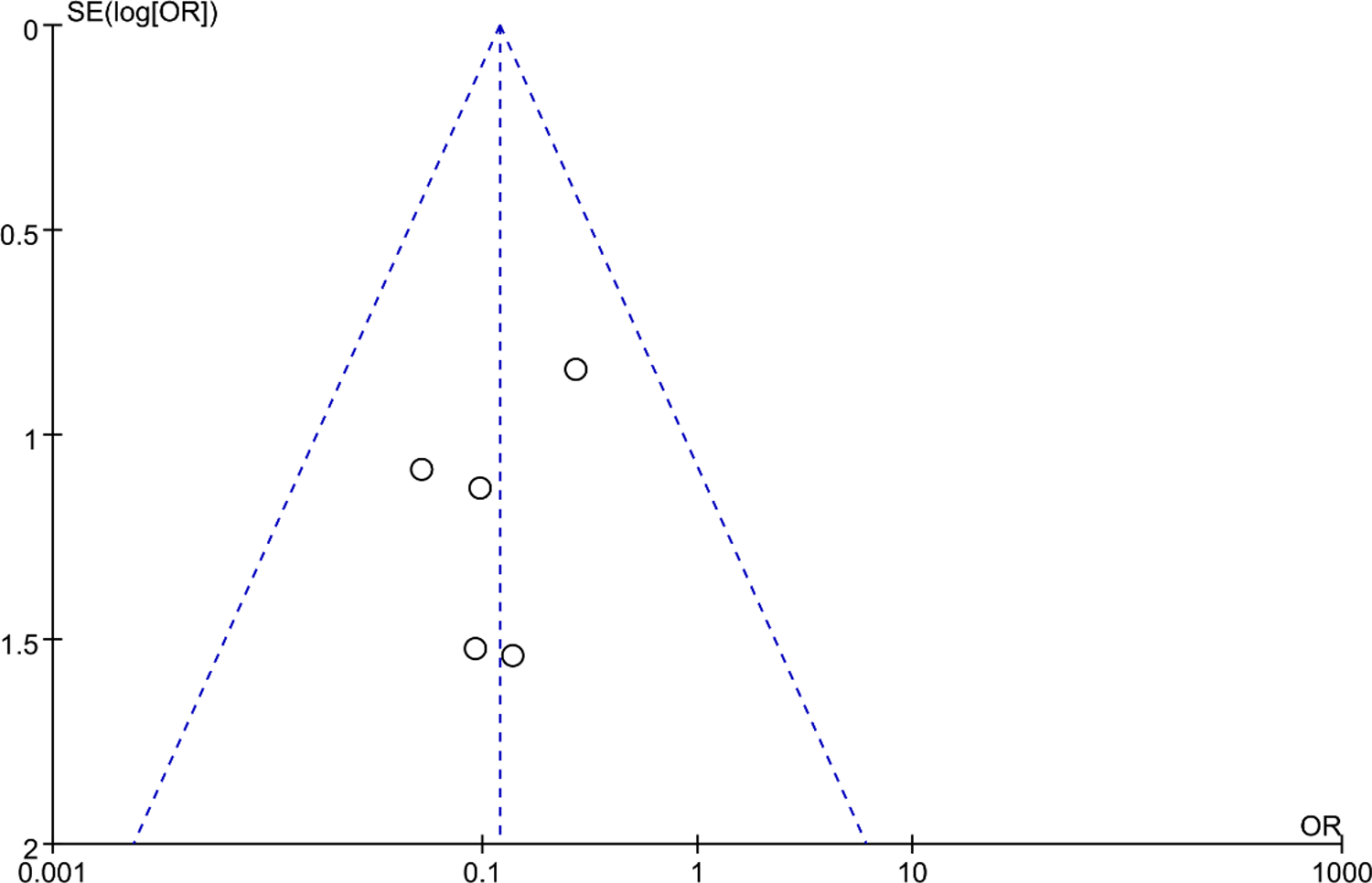
Funnel plot of severe subsidence of vertebral body.

**Figure 13:**
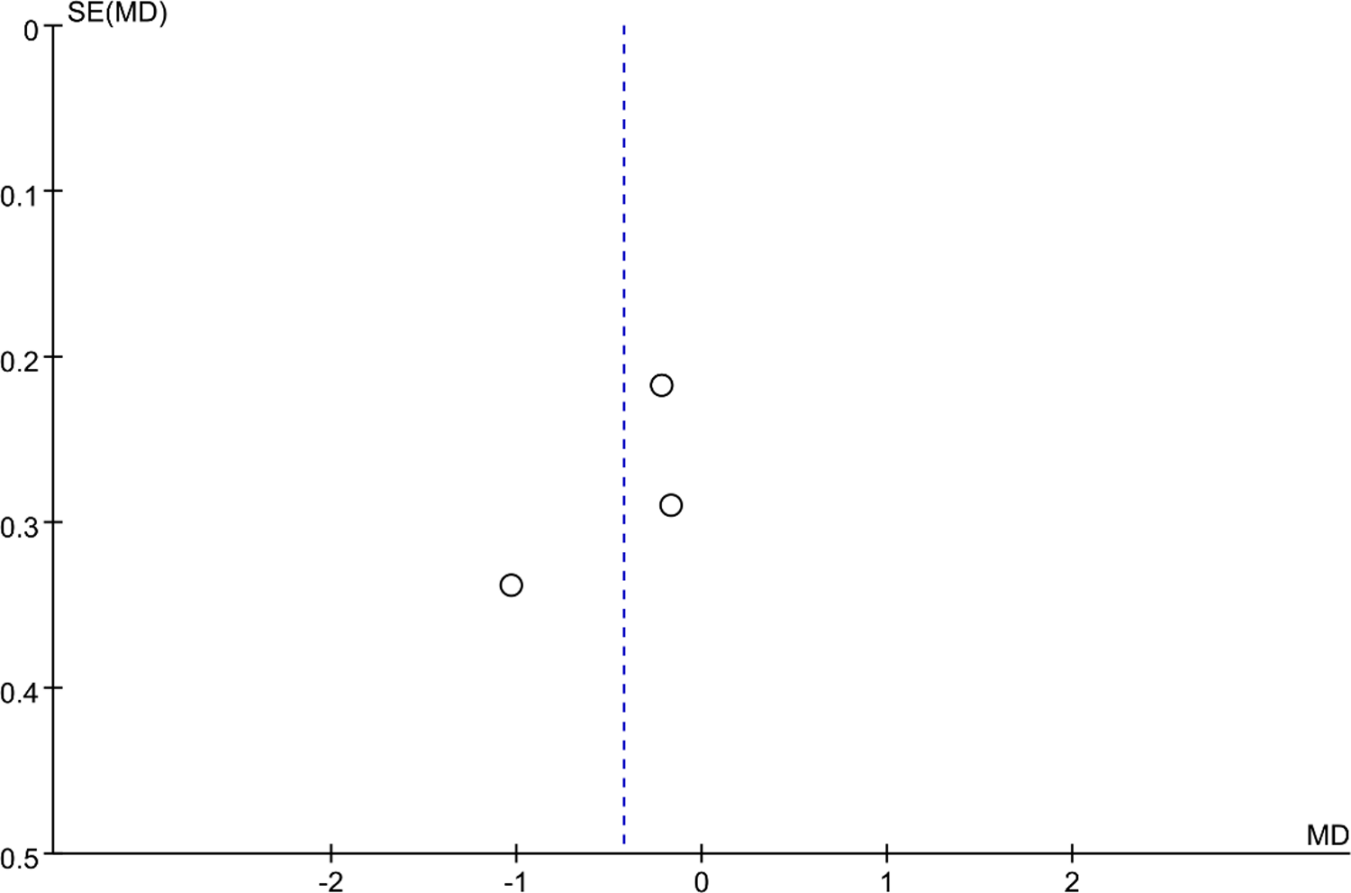
Funnel plot of final follow-up Visual Analog Scale (VAS) scores.

**Figure 14:**
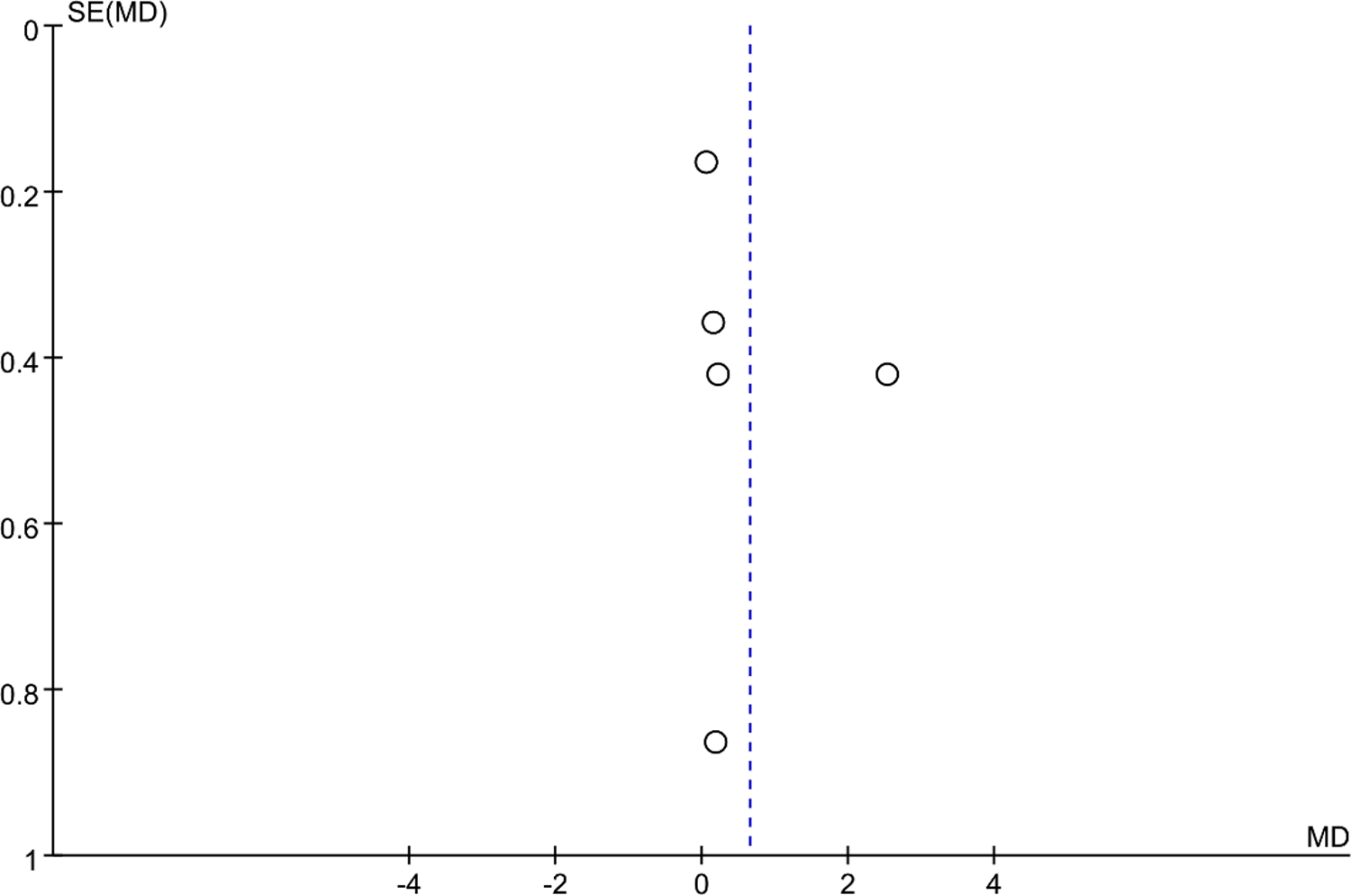
Funnel plot of final follow-up Japanese Orthopedic Association (JOA) scores.

**Figure 15:**
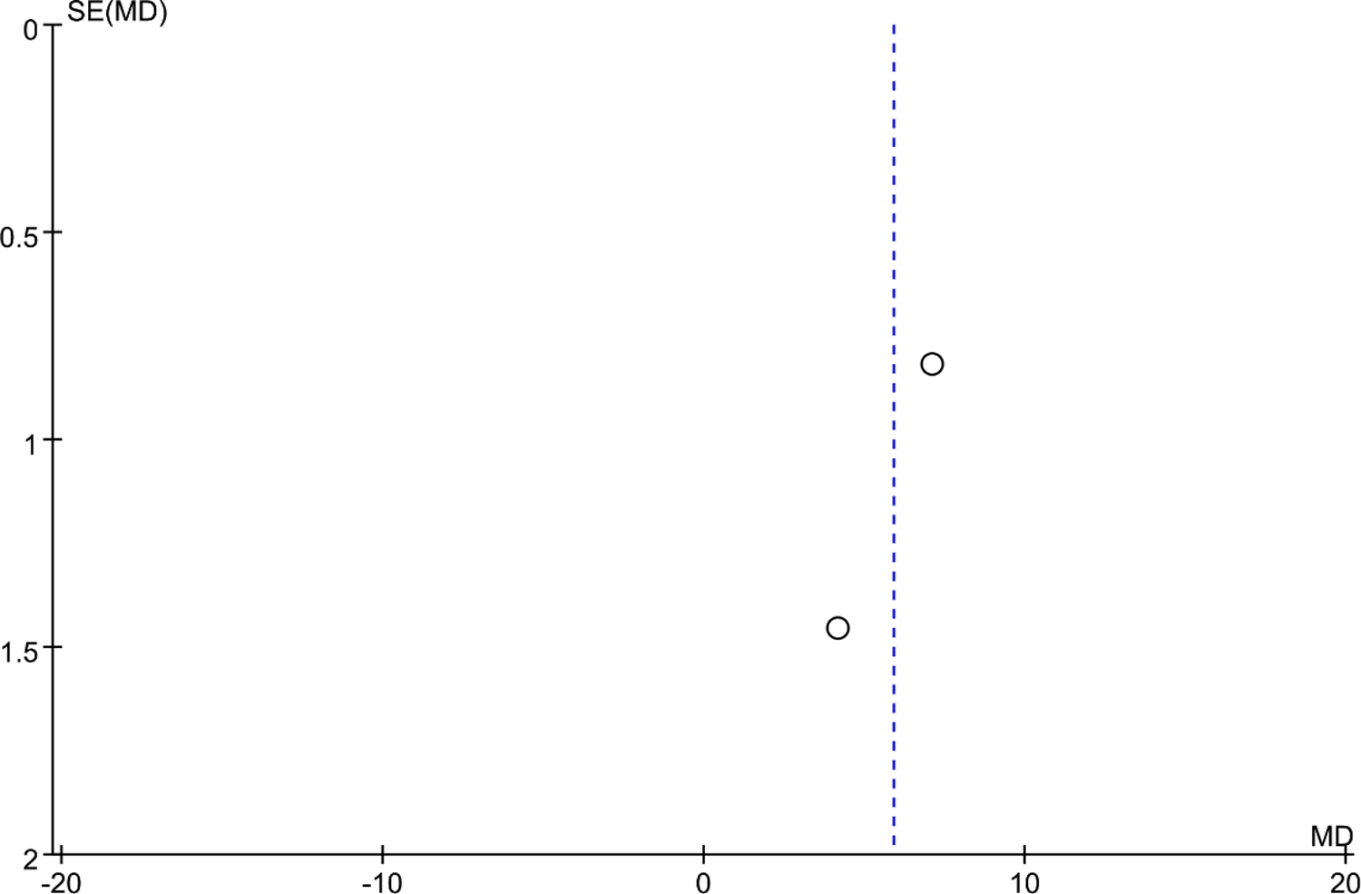
Funnel plot of final follow-up cervical C2–C7 Cobb angle.

Funnel plots were constructed to assess publication bias, and the results were largely symmetrical, indicating acceptable publication bias in our analysis. However, the distribution of JOA scores was significantly asymmetric at the last follow-up, suggesting that publication bias is likely (Figures 9–15).

## Discussion

Anterior cervical corpectomy and fusion(ACCF) is a common surgical method to treat cervical-related diseases (5, 10), Spinal surgeons are always looking for a more efficient and more secure implants and Titanium Mesh Cage(TMC) has gradually replaced autologous bone graft and become mainstream (15, 27, 28), the emergence of Three-dimensional printing technology provides a brand-new choice of fusion implant (29), However, at present, there is no large-scale research to prove whether 3D printed artificial vertebral body is more safe and effective than traditional TMC. In this paper, six cohort studies were included to analyze the efficacy and safety of 3D-printed artificial vertebral body in single-level anterior cervical corpectomy and fusion(SL-ACCF), and the results show that 3D printing artificial vertebral body is superior to traditional TMC in shortening the operation time, reducing the occurrence of Severe subsidence of vertebral body and restoring C2–C7 Cobb Angle, but there is no significant difference in Japanese Orthopedic Association (JOA) scores, Visual Analog Scale (VAS) scores and reducing intraoperative blood loss.

During the traditional operation, the operator repeatedly tried to select a suitable interbody fusion cage, it increases the risk of fracture of steel plate that is too large, and loosening and displacement are easy to occur when it is too small, thus increasing the operation time (30). While the 3D-printed artificial vertebral body has been modeled by the CT or MRI spiral scanner of the whole vertebral body, and the final height of the vertebral body of the fusion cage is almost the same as the anatomical height of the patient, thus avoiding the repeated selection of the appropriate interbody fusion cage and shortening the operation time (31).

After the surgery, vertebral body height and cervical sagittal balance has always been the focus of clinical doctors, traditional TMC is easy to decrease the height of vertebral body after operation, especially when TMC subsides, it is easy to decrease the height of intervertebral foramen and the number of wrinkles of posterior longitudinal ligament, which leads to the symptoms of nerve and spinal cord compression (9, 32, 33). The main reason for these situations is that the elastic modulus of the TMC is inconsistent with that of normal human bones, resulting in stress shielding. The 3D-printed artificial vertebral body with upper dome structure and lower inclination Angle designed by Lu (16) et al. can increase the contact area with the upper and lower vertebral bodies, reducing the concentrated stress and the drop of vertebral body height. Studies have shown that the porosity of the implant should be controlled between 60% and 80% to balance the elastic modulus and compression strength of the prosthesis. Rapuano (34) et al. confirmed that cells spread well on the surface of porous titanium alloy, and the micro-rough structure was beneficial to the aggregation and growth of bone cells *in vitro* and Olivares-Navarrete (35) et al. also found that the design of porous rough titanium alloy can create an osteogenic environment containing bone morphogenetic protein 2(BMP2), BMP4 and BMP7, promote the differentiation and maturation of osteoblasts (36), and facilitate the earlier completion of osseous fusion and the stability of implants (19). Cervical C2–C7 Cobb Angle is an important metric to measure the sagittal balance of the cervical spine. Also, due to the rational structure of the upper and lower ends of the 3D-printed artificial vertebral body, reasonable porous design, elastic modulus to that of normal bone, and providing a microenvironment for the growth of bone cells, the cervical C2–C7 Cobb Angle outperforms better than that of the titanium cage. This study also confirmed the advantages of 3D-printed vertebral body in reconstruction of the anterior column and restoring the sagittal balance of the cervical spine.

## Limitations

First of all, there were no randomized controlled trials in all the included studies, but no serious bias was found in the published bias test [Figures 9–15]. Second, there are differences in the 3D printed implants used in most studies, such as porosity, upper and lower structures, etc. There is a high degree of heterogeneity among the various studies, such as the operation time, but after excluding each study sequentially, the recalculated pooled results did not significantly change, indicating that there was no outlying study that significantly influenced the overall results. All the articles included are from China, and most of the samples are Asian, so they are not well represented. Only 6 articles were included, and the sample size is small, so a large number of randomized controlled trials with long follow-up times are needed to complement the conclusions.

## Conclusion

This meta-analysis demonstrated that 3D-printed artificial vertebral body is superior to traditional TMC in reducing the operation time and maintaining the postoperative vertebral height and restoring sagittal balance of cervical spine in single-level anterior cervical corpectomy and fusion.

## Data Availability

The original contributions presented in the study are included in the article/Supplementary Material, further inquiries can be directed to the corresponding author/s.
